# Editorial: Advances in tomato and tomato compounds research and technology

**DOI:** 10.3389/fnut.2022.1018498

**Published:** 2022-08-31

**Authors:** José Pinela, Spyridon A. Petropoulos, Lillian Barros

**Affiliations:** ^1^Centro de Investigação de Montanha (CIMO), Instituto Politécnico de Bragança, Bragança, Portugal; ^2^Laboratório Associado para a Sustentabilidade e Tecnologia em Regiões de Montanha (SusTEC), Instituto Politécnico de Bragança, Bragança, Portugal; ^3^Department of Agriculture Crop Production and Rural Environment, University of Thessaly, Volos, Greece

**Keywords:** *Solanum lycopersicum* L., nutritional quality, flavor quality, processing, lycopene, phenolic compounds, bioaccessibility, health benefits

Tomato is the fruit of *Solanum lycopersicum* L., a Solanaceae crop of worldwide economic importance. Today, there are a large number of tomato cultivars and local varieties with different morphological and sensory characteristics, as well as a wide range of tomato-based foods. These are great dietary sources of micronutrients and bioactive compounds, such as lycopene, vitamins, minerals, and phenolic compounds, which have been linked to many health-promoting effects (1). Several pre- and postharvest efforts have been made to improve the quality of tomato fruit and derived food products, as both tomato production and processing are being carried out under more sustainable and innovative practices. This Research Topic features 12 papers covering relevant subjects, including the production and processing of tomatoes and tomato-based foods and ingredients, as well as the bioaccessibility and health-promoting effects of tomato bioactive compounds.

Traditional varieties represent an important component of agricultural biodiversity and play a vital role in the sustainability and security of the agri-food system (2). In this sense, Raigón et al. characterized morphological, nutritional, and chemical characteristics of two Malacara tomato cultivars (with red and yellow fruits) grown under organic farming conditions. This type of cultivars (“Cuelga”) originates from Sierra de Cádiz, Spain, is cultivated and harvested during the summer and tomato trusses are hung from beams in the farmhouses for consumption during the winter; hence the name “Cuelga” which stands for hanging. The main differences among these small, pallid tomatoes were mainly related to morphological parameters, but also to fiber, minerals (Fe, Mg, Ca), and lycopene contents. 2-Phenylethanol was detected in both Malacara cultivars, and the low concentration of aldehydes in this varietal type could be related to its long shelf-life.

The effect of different production systems on tomato quality was also addressed in this Research Topic. Ilahy et al. investigated the impact of pre-harvest treatments with saline water and spent engine oil on nutritional quality of ripe tomatoes. Moderate salinity stress promoted an increase in soluble solids, lycopene, total phenolics, and radical scavenging activity compared with the control treatment (untreated plants). In turn, the flavonoid content decreased when plants received the treatment of 0.5% spent engine oil. Interestingly, the correlation of the redness/yellowness ratio with β-carotene, lycopene, vitamin C, tocopherols, and radical scavenging activity was suggested as a possible indicator of tomato fruit quality in areas inflicted by such agro-environmental restrictions. In another study, Erika et al. analyzed sensorial properties and volatile organic compounds (VOCs) associated with tomato flavor under organic low-input production systems. Salad and cocktail cultivars showed a wide range of variation for the studied traits, with the exception of specific VOCs. Twelve VOCs were correlated with sensorial attributes and allowed the differentiation of the cultivars depending on their fruit types, namely salad and cocktail cultivars. Among these, phenylethyl alcohol and benzyl alcohol were positively correlated with the acceptability of cocktail cultivars, whereas 2-isobuthylthiazole and 6-methyl-5-hepten-2-ol negatively was correlated with the acceptability of salad cultivars. Therefore, organic breeders were recommended to use cultivars from a wide range of breeding programs to improve important tomato quality and agronomic traits and compromise the trade-off of high yield and quality.

Light-emitting diode (LED) lamps are increasingly being used in tomato production systems. Alsina et al. evaluated the effect of additional lighting of different quality used in greenhouse cropping systems on the accumulation of bioactive compounds in tomatoes. High-pressure sodium lamps (HPSL) stimulated the accumulation of primary metabolites; the soluble solids content was higher compared to other lighting sources. Since LED and induction lamps emit about 20% blue-violet light, the obtained results suggested that blue-violet light of the spectrum stimulates the accumulation of phenolic compounds in tomatoes when additional lighting from these lights sources is implemented. Moreover, red fruit varieties tend to synthesize more β-carotene under these light sources, compared to HPSL, while the increase of blue light promoted the synthesis of lycopene, phenolics, and flavonoids and decreased soluble solids content. In the same context, Wang et al. studied the suitability of red and blue LED for supplementing light on tomato plants for different time periods in the morning and evening. The accumulation of vitamin C, organic acids, amino acids, carotenoids, phenolic acids, and other health-promoting compounds in fruit was promoted when plants were treated with light supplementation in the morning, while light supplementation in the evening increased the contents of sugars, flavonoids, and aromatic compounds. Thus, it could be suggested that morning light supplementation may improve the nutritional quality of tomato fruit, while evening treatments are beneficial to their flavor-related parameters.

The bioactive constituents of tomato fruit are affected by several factors, including genetic features, environmental conditions, maturation degree, and postharvest treatments. In this sense, Lima et al. performed a literature review aiming to investigate how pre- and postharvest factors may influence the content of bioactive compounds in tomatoes (with a particular focus on phenolic compounds, carotenoids, and biogenic amines) and how some heat processing methods may change the antioxidant status of food products. The potential for reintroducing tomato by-products into the value cycle was also addressed in this mini-review.

This Research Topic also covered important findings for the tomato processing and trade sectors. A non-destructive method for estimating soluble solids and lycopene contents in tomato fruit or for rapid analysis of tomato homogenates during raw material quality assessment was developed by Égei et al. using visible and near-infrared (Vis-NIR) absorbance and reflectance data. In turn, tomatoes at the mature-red and mature-green stages are prone to chilling injury when stored at temperatures below 5 and 10°C, respectively, leading to a decline in quality and shelf-life, thus restricting trade flexibility. Zhao et al. reported that the silencing of Sly-miR171e enhanced the expression of *GRAS24* (the target gene of miR171), increased the gibberellic acid content and the expression of *CBF1* and *COR* genes, and by which chilling injury of tomato fruit was alleviated. In the study by Zhang et al., lycopene was successfully encapsulated in polyelectrolyte complex nanoparticles made with a negatively charged polysaccharide and positively charged sodium caseinate. These stable nanoparticles exhibited improved water-solubility, powerful antioxidant capacity, and controlled release ability through a simulated gastrointestinal tract when compared with free lycopene. Furthermore, these biocompatible nanoparticles increased cell viability, prevented apoptosis and protected cells from oxidative damage, thus constituting a potential health supplement or nutraceutical to improve human health. In a study with canned tomatoes, Izzo et al. showed that a noticeable percentage of rutin, naringenin, chlorogenic acid, and lycopene remains bioaccessible after simulated gastrointestinal digestion, thus evidencing which compounds may exert beneficial effects on consumers' health.

Regarding the health benefits of tomato fruit and tomato compounds, Cámara et al. revised the scientific evidence regarding the beneficial effects of tomato products on both cardiovascular disease prevention and antiplatelet aggregation, as well as the European Food Safety Authority health claims for tomato products. Curiously, only one health claim has been approved so far for a water-soluble concentrated extract of tomato, namely “helping to maintain normal platelet aggregation, which contributes to healthy blood flow.” Finally, Huang et al. concluded that lycopene can effectively alleviate liver steatosis induced by a high-fat diet and could be used as a possible dietary strategy for the control and treatment of non-alcoholic fatty liver disease. This beneficial effect was related to the fact that lycopene increased the expression of genes related to liver lipid metabolic process.

Overall, this Research Topic showed that tomato is a functional food which remains in the spotlight of many researchers who focus on different nutritional/nutraceutical quality issues, ranging from its production to the final impact on consumers' health. The findings compiled in the present Research Topic highlight the importance of scientific evidence regarding the health effects of tomato fruit and food products and light up new directions for further research.

## Author contributions

All authors listed have made a substantial, direct, and intellectual contribution to the work and approved it for publication.

## Funding

The authors are grateful to the Foundation for Science and Technology (FCT, Portugal) for financial support through national funds FCT/MCTES (PIDDAC) to CIMO (UIDB/00690/2020 and UIDP/00690/2020), SusTEC (LA/P/0007/2021), and to FCT for the contracts of JP (CEECIND/01011/2018) and LB (CEEC Institutional).

## Conflict of interest

The authors declare that the research was conducted in the absence of any commercial or financial relationships that could be construed as a potential conflict of interest.

## Publisher's note

All claims expressed in this article are solely those of the authors and do not necessarily represent those of their affiliated organizations, or those of the publisher, the editors and the reviewers. Any product that may be evaluated in this article, or claim that may be made by its manufacturer, is not guaranteed or endorsed by the publisher.
